# PriaXplore^® ^- a novel technology platform for the identification of small molecule modulators of protein-protein interactions

**DOI:** 10.1186/1758-2946-5-S1-P35

**Published:** 2013-03-22

**Authors:** Susanne Eyrisch, Tobias Girschick, Günter Ross, Cédric Kalinski, Vladimir Khazak, Lutz Weber

**Affiliations:** 1 Priaxon AG, Munich, 81379, Germany

## 

Protein-protein interactions (PPIs) are broadly recognized as an emerging target class for a whole new era in drug discovery. They show significant differences to classical target classes and have been regarded as "undruggable" for a long time. Priaxon AG has developed a unique, proprietary technology platform for PPI drug discovery. PriaXplore^® ^employs novel computational and synthetic approaches to address PPIs with small molecule modulators. PriaXplore^® ^utilizes Multicomponent Reactions (MCRs) to create an *in silico *search space of 200 million compounds pre-selected for PPI relevance. The PriaXplore^® ^*in silico *screening process combines independent descriptor systems to search the MCR product space with maximum speed and accuracy. This combination of independent description methods enables a highly efficient selection of potential hit compounds that are further filter by our in-house docking protocol PriaDock. As protein-protein interaction sites are known to be very flexible and binding pockets of small molecule modulators may not be (fully) accessible in all protein structures, PriaDock uses multiple protein conformations derived from crystal/NMR structures or molecular dynamics simulations to account for this flexibility. Thus, smaller sets of compounds have to be synthesized and tested, lab resources are saved.

PriaXplore^® ^has been validated by a successfully out-licensed Mdm2/p53 PPI inhibitor program. Currently PriaXplore^® ^is applied in several drug discovery projects which are run either as Priaxon in-house projects or as cooperations with Pharma partners.

